# Organic Microbial Electrochemical Transistor Monitoring Extracellular Electron Transfer

**DOI:** 10.1002/advs.202000641

**Published:** 2020-06-09

**Authors:** Gábor Méhes, Arghyamalya Roy, Xenofon Strakosas, Magnus Berggren, Eleni Stavrinidou, Daniel T. Simon

**Affiliations:** ^1^ Laboratory of Organic Electronics Department of Science and Technology Linköping University Norrköping 60174 Sweden; ^2^ Wallenberg Wood Science Center Department of Science and Technology Linköping University Norrköping 60174 Sweden

**Keywords:** extracellular electron transfer, microbial electrochemical systems, organic electrochemical transistors (OECTs), PEDOT:PSS, *Shewanella oneidensis*

## Abstract

Extracellular electron transfer (EET) denotes the process of microbial respiration with electron transfer to extracellular acceptors and has been exploited in a range of microbial electrochemical systems (MESs). To further understand EET and to optimize the performance of MESs, a better understanding of the dynamics at the microscale is needed. However, the real‐time monitoring of EET at high spatiotemporal resolution would require sophisticated signal amplification. To amplify local EET signals, a miniaturized bioelectronic device, the so‐called organic microbial electrochemical transistor (OMECT), is developed, which includes *Shewanella oneidensis* MR‐1 integrated onto organic electrochemical transistors comprising poly(3,4‐ethylenedioxythiophene):poly(styrenesulfonate) (PEDOT:PSS) combined with poly(vinyl alcohol) (PVA). Bacteria are attached to the gate of the transistor by a chronoamperometric method and the successful attachment is confirmed by fluorescence microscopy. Monitoring EET with the OMECT configuration is achieved due to the inherent amplification of the transistor, revealing fast time‐responses to lactate. The limits of detection when using microfabricated gates as charge collectors are also investigated. The work is a first step toward understanding and monitoring EET in highly confined spaces via microfabricated organic electronic devices, and it can be of importance to study exoelectrogens in microenvironments, such as those of the human microbiome.

## Graphical abstract

The real‐time monitoring of extracellular electron transfer at the microscale is demonstrated with rapid response to lactate, without sophisticated signal amplification. The miniaturized bioelectronic device includes *Shewanella oneidensis* MR‐1 integrated onto organic electrochemical transistors comprising poly(3,4‐ethylenedioxythiophene):poly (styrenesulfonate) (PEDOT:PSS) and poly(vinyl alcohol) (PVA). The work can be important to study exoelectrogens in microenvironments, such as in the human microbiome.

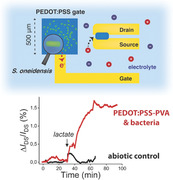

Bacteria living in oxygen‐limited environments, such as *Shewanella oneidensis* MR‐1 (*S. oneidensis* MR‐1, where MR‐1 stands for "manganese reducer"), complement the absence of oxygen in their metabolic cycle by extracellular electron transfer (EET). In this process, typically metal oxides or even polarized electrodes take the role of terminal‐end electron acceptors instead of oxygen. EET is important for a wide variety of industrial applications including energy generation via microbial fuel cells (MFCs),^[^ ^1^ ^]^ biobatteries,^[^ ^2^ ^]^ and whole cell‐based biophotovoltaic cells (BPVCs),^[^ ^3^ ^]^ storing electrical energy in chemical bonds (microbial electrosynthesis),^[^ ^4^ ^]^ detection of analytes,^[^ ^5^, ^6^ ^]^ or even cost‐efficient preparation of graphene from graphene oxide^[^ ^7^ ^]^ in microbial electrochemical systems (MESs).^[^ ^8^ ^]^ From the perspective of human health, EET has recently been implicated in colonization of the human gut by pathogenic bacteria.^[^ ^9^, ^10^ ^]^ EET has also been discussed as an extraterrestrial metabolism that theoretically may support life on iron‐rich planets such as Mars.^[^ ^11^ ^]^ Monitoring EET under various conditions is thus important for a range of industrial, agricultural, and medical processes.

The most common methods for monitoring microbial EET include a variety of electrochemical techniques in which the metabolism of bacteria can be monitored by collecting a current of electrons that are transferred to a working electrode (WE) during the oxidation of lactate^[^ ^12^, ^13^, ^14^, ^15^, ^16^ ^]^ or other substrates in anaerobic conditions. To improve the efficiency of electron transfer, bio‐hybrid electrodes have recently been demonstrated by combining living bacteria into the bulk of conducting polymer films based on poly(3,4‐ethylenedioxythiophene):poly(styrenesulfonate) (PEDOT:PSS), thus minimizing the distance and increasing the interface area between bacteria and electrode.^[^ ^17^ ^]^ The output current in such configurations is proportional to the number of bacteria, their metabolic activity, and the electrode area/volume. However, the amount of current collected by an electrode as part of EET is typically ≈10–100 fA per bacterial cell.^[^ ^18^, ^19^ ^]^ Thus, electrodes with large surface area and high concentration of bacteria are necessary for a substantial output signal. In contrast, when monitoring of local events or low number of bacteria is needed, this electrode setup is of limited use due to its bulky nature. Moreover, scaling down electrode dimensions typically results in lower output currents, thus reducing the signal‐to‐noise ratio. For such scenarios, bulky or expensive equipment is needed to amplify the signal above the noise level.

Organic electrochemical transistors (OECTs), owing to their inherent amplification properties, are able to locally improve the monitoring of weak biological signals. OECTs have been demonstrated in a wide assortment of bioelectronic applications such as enzymatic sensing,^[^ ^20^ ^]^ monitoring of cell integrity,^[^ ^21^, ^22^ ^]^ and recording of neuronal activity.^[^ ^23^ ^]^ Recently, we utilized an OECT functionalized by glucose oxidase and Pt nanoparticles to monitor glucose export from plant chloroplasts in real time.^[^ ^24^ ^]^ The high transconductance of OECTs,^[^ ^25^ ^]^ i.e., the transduction of low ionic or electrochemical signals at the gate to large electronic currents at the channel, makes them perfect candidates to monitor local events such as cell or bacteria metabolism. Moreover, microfabrication techniques are readily available to create micrometer‐size OECTs, suitable as interfaces with single cells or cell clusters.^[^ ^26^ ^]^ Indeed, OECTs have been proposed as disposable sensors for the detection of *Escherichia coli*. However, such detection was based on electrostatic interactions between the cells and the transistor, where bacteria were attached to the PEDOT:PSS channel by antibody‐binding.^[^ ^27^, ^28^ ^]^

In this work, we developed an organic microbial electrochemical transistor (OMECT) to monitor EET. The OMECT consists of a microfabricated OECT with a PEDOT:PSS channel and a gate electrode comprising PEDOT:PSS integrated with *S. oneidensis* MR‐1. To encourage bacterial attachment to the gate electrode (surface area only 0.25 mm^2^), we defined and connected the OECT gate as the positively biased WE, thus enabling it to serve as an electron sink for the bacterial lactate metabolism under anaerobic conditions. Moreover, by utilizing a blend of PEDOT:PSS with poly(vinyl alcohol) (PVA) we observed a higher level of EET as compared to using PEDOT:PSS as the only active material in the channel and the gate.^[^ ^29^ ^]^ Fluorescence images validate the presence of bacteria on the gate of the OECTs. Interestingly, classical electrochemical techniques, including electrochemical impedance spectroscopy (EIS), proved insufficient in detecting EET signatures from these low number of bacteria that could fit on the 0.25 mm^2^ gate area when operated in two‐electrode configuration. However, in transistor configuration, we succeeded in recording temporal lactate response signals by transducing and amplifying gate‐to‐drain currents. The OMECT concept can be used in similar applications where optimizing and understanding bacterial metabolism is of interest, or to specifically optimize MFCs and MESs.

The setup of an OMECT consisted of a planar OECT comprising M9 buffer solution as electrolyte, confined with a glass/plastic well. Nitrogen gas was introduced to the system to maintain anaerobic conditions during the attachment of the bacteria and during lactate sensing (**Figure** **1**a). We fabricated OECTs using photolithographic processes and Parylene C (Pa‐C) as an insulating and sacrificial layer, as previously reported.^[^ ^30^ ^]^ The OECTs had square‐shaped gates of 500 × 500 µm^2^ and a channel of 100 µm width and 10 µm length (Figure 1a,b). This OECT configuration is known to exhibit high transconductance.^[^ ^31^, ^32^ ^]^ To investigate the adhesion characteristics of negatively charged *S. oneidensis* MR‐1 on the gate, we employed devices with two formulations; one with PEDOT:PSS and one with PEDOT:PSS‐PVA as the active material system on top of a gold layer, where PVA is expected to facilitate the attachment of bacterial cells to the surface of PEDOT:PSS films.^[^ ^29^ ^]^ The working principle of the OMECT relies on *S. oneidensis* MR‐1, attached onto the gate, metabolizing lactate under anoxygenic conditions and thereby transferring electrons to the gate as a metabolic “byproduct.” In this case, the gate plays the role of electron sink for the microbial respiration, a function normally (in oxygenic environments) fulfilled by oxygen. To maintain electroneutrality, cations are expelled from the electrolyte and enter the OECT channel at the same time as the gate receives charges from the bacteria (Figure 1c). This effect leads to a partial de‐doping of PEDOT:PSS, decreasing its conductivity that in turn decreases the drain–source current (*I*_DS_).

**Figure 1:**
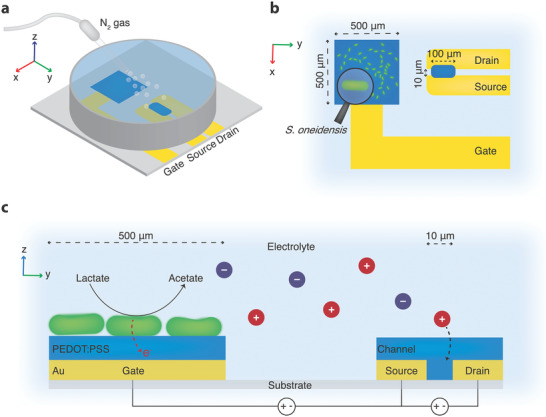
Schematic illustrations showing the operating principle of the OMECT. a) Overview of the device and operation. b) Magnified top view of the gate covered by *S. oneidensis* MR‐1 and channel area. c) Cross‐sectional view of the gate and channel including the mechanism of lactate metabolism by bacteria and OECT operation. Spheres with + and − signs represent positive and negative ions, respectively, e^−^ represents electrons. Bacteria (green rods) and thickness of Pa‐C are not to scale.

For the attachment of bacteria on the gate of the OECT we used a chronoamperometric technique. A glass/plastic well, attached to the substrate supporting the device, contained a mixture of M9 buffer, excess of bacteria (*S. oneidensis* MR‐1, optical density at 600 nm (OD_600_) = 1.56) and excess of carbon source (80 × 10^−3^ m lactate) in a total volume of 1.75 mL. To permit visualization of the bacteria via fluorescence imaging, the *S. oneidensis* MR‐1 strain expressing green fluorescent protein (GFP) was used.^[^ ^33^ ^]^ The gate of the OECT served as the WE and a Ag/AgCl wire inserted into the solution was used as a combined counter and reference electrode in a two‐electrode setup. The solution was deoxygenated by continuous purging with nitrogen before and during the experiments. A constant voltage of *V*_G_ = +0.3 V_Ag/AgCl_ was applied to the gate and the current was recorded. **Figure** **2**a shows that the gate current constantly increased over a period of ≈13 h, finally reaching ≈35 nA for PEDOT:PSS and ≈75 nA for PEDOT:PSS‐PVA. These currents correspond to the transfer of charges from bacteria to the collecting gate electrode. We anticipate no microbial growth during the course of the current recordings that could influence the signals as it was found earlier that *S. oneidensis* MR‐1 does not grow aerobically in M9 solution supplemented with only lactate.^[^ ^17^ ^]^ We recorded currents below 1 nA for both electrodes when the EET was inhibited by purging the solution with oxygen instead of nitrogen, see Figure 2a. Likewise, we recorded the same low current level when employing the Δ*mtrB* mutant, which cannot perform EET, in a PEDOT:PSS‐PVA device under nitrogen purging (Figure 2a). Finally, the abiotic chronoamperometric experiments performed with lactate in solution but no bacteria in the system produced negligible signals for both electrodes (Figure 2a). These observations indicate that bacterial EET into the PEDOT:PSS and PEDOT:PSS‐PVA gates is the source of the recorded currents, confirming previous observations that *S. oneidensis* MR‐1 can use multiple EET mechanisms to reduce PEDOT:PSS.^[^ ^17^ ^]^ In most experiments, the chronoamperometry was interrupted after ≈4 h because good attachment of bacteria to the gate had already occurred at this time.

**Figure 2:**
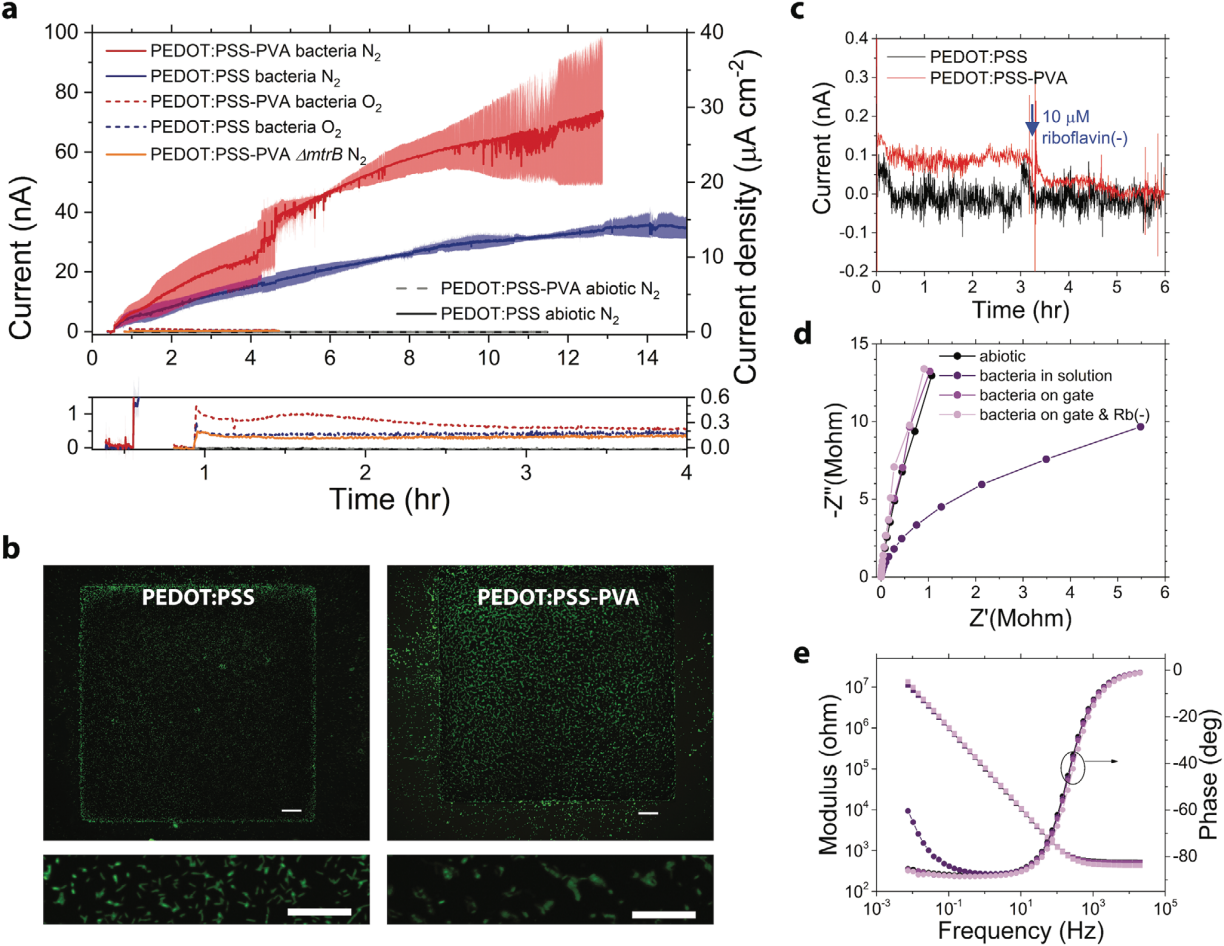
Attachment of *S. oneidensis* MR‐1 bacteria on OECT gate and evaluation of metabolic activity. a) Chronoamperometric curves showing EET arising from lactate metabolism with excess of bacteria (OD_600_ = 1.56) in the electrolyte M9 with added 80 × 10^−3^ m lactate, at +0.3 V_Ag/AgCl_ on gate. GFP mutant in anoxygenic (solid blue and red lines, respectively), oxygenic (dashed blue and red lines, respectively) environments; Δ*mtrB* mutant on PEDOT:PSS‐PVA device (solid orange line); and abiotic devices (solid black and dashed gray lines, respectively) in anoxygenic environment. Bottom graph is a magnified view of the low signals during the first ≈3 h. Solid blue and red lines are mean curves and translucent ribbons are standard deviation (SD) for PEDOT:PSS *n* = 6 (up to 4.3 h), *n* = 3 (4.3–8 h), *n* = 2 (from 8 h) and for PEDOT:PSS‐PVA *n* = 6 (up to 4.2 h), *n* = 3 (4.2–4.6 h), *n* = 2 (from 4.6 h). b) Fluorescence FITC microscopy images of PEDOT:PSS and PEDOT:PSS‐PVA gates after chronoamperometric attachment of GFP bacteria. Scale bars on top and bottom images are 50 and 20 µm, respectively. The full version of the bottom images can be found as Figure S1 (Supporting Information). c) Chronoamperometric currents after attachment with the same conditions as in (a) but without free‐flowing bacteria in the bulk, i.e., bacteria only on gate. Blue arrow indicates the addition of 10 × 10^−6^ m Rb(−). Curves were smoothed to reduce noise. d) Nyquist and e) Bode plots as representative electrochemical impedance spectrographs of the PEDOT:PSS‐PVA gate in M9 and 80 × 10^−3^ m lactate before the addition of bacteria (“abiotic”), after ≈3 h of chronoamperometric attachment with bacteria at OD_600_ = 1.56 (“bacteria in solution”), ≈3 h after exchanging the solution to M9 and 80 × 10^−3^ m lactate (“bacteria on gate”), and ≈3 h after the addition of Rb(−) (“bacteria on gate & Rb(−)”). All EIS measurements were carried out at *V*_G_ = +0.3 V_Ag/AgCl_ to maintain bacterial EET.

Attachment of bacteria on the surface of PEDOT:PSS or PEDOT:PSS‐PVA gates was confirmed by fluorescence and bright field microscopies. Prior to imaging, the devices were processed by a gentle washing step with M9 solution and deionized water, followed by a short drying step with nitrogen gas. The PEDOT:PSS and PEDOT:PSS‐PVA gates, previously connected as working electrodes, showed clear fluorescence signals from bacteria when imaged through a suitable filter set (Figure 2b and Figure S1, Supporting Information) for GFP. The bacteria covered most of the gate area, forming relatively larger clusters on PEDOT:PSS‐PVA as compared to PEDOT:PSS; the length of individual cells is 2.0 ± 0.2 µm, estimated from nine randomly selected cells on a PEDOT:PSS gate (Figure S2, Supporting Information). The number of cells on the PEDOT:PSS gate was *n* ≈ 18 000, corresponding to the density of *d* ≈ 72 000 mm^−2^ or ≈ 1 cell/14 µm^2^, estimated by ImageJ. This number is at least 5 orders of magnitude lower compared to the expected >10^9^ mL^−1^ of cells at OD_600_ = 1.56 during the chronoamperometric attachment (volume 1.75 mL), based on estimations with *E. coli*.^[^ ^34^ ^]^ For the PEDOT:PSS‐PVA gate the number of bacteria could not be estimated correctly due to the larger clusters present. On the other hand, we detected significantly lower fluorescence from the PEDOT:PSS control gate that was also present in the electrolyte (and therefore exposed to bacteria in solution) but was not biased during the chronoamperometric attachment. The PEDOT:PSS‐PVA control gate, however, did exhibit a bright fluorescence signal (Figure S3, Supporting Information). These observations are consistent with bright field images, where bacteria appear as bright regions on the gray background (Figure S4, Supporting Information). According to earlier studies, species of *Shewanella* MR‐1 tend to self‐attach to carbon based electrodes via hydrophobic anchoring from outer membrane lipoproteins.^[^ ^35^, ^36^ ^]^ Consequently, the differences in bacterial coverage between the two electrodes might be attributed to reduced anchoring for PEDOT:PSS films, presumably due to the excess of hydrophilic PSS present on the surface of PEDOT:PSS, and enhanced binding to the PEDOT:PSS‐PVA films, presumably due to hydroxyl groups at the PVA surface.^[^ ^29^ ^]^ Finally, by using bright field microscopy we also confirmed the presence of the Δ*mtrB* mutant on both biased and control PEDOT:PSS‐PVA gates, the former corresponding to the data shown in Figure 2a (Figure S5, Supporting Information). This mutant shows a significant aggregation on both gates with larger clusters formed on the unbiased control gate.

It is well known that *S. oneidensis* MR‐1 carries out EET simultaneously via a direct route of physical contact to electrodes by c‐type cytochromes located in its outer membrane, and also via indirect endogenous flavin shuttles. These direct and indirect mechanisms typically account for 20–30% and 80–70% of the total metabolic currents, respectively.^[^ ^12^, ^37^ ^]^ Therefore, metabolic currents of both PEDOT:PSS and PEDOT:PSS‐PVA devices, described above, may predominantly originate from the highly concentrated nonattached bacteria in the bulk, performing EET via the indirect flavin‐mediated route. To reveal the extent of contribution from these planktonic cells, we repeated the chronoamperometric experiments in both types of devices after gently washing an OECT where bacteria were already attached to the gate only, with the same conditions (including lactate) but without the addition of excess of free‐flowing bacteria. Surprisingly, the resulting signals showed negligible currents for both PEDOT:PSS and PEDOT:PSS‐PVA systems (Figure 2c), indicating that the previously observed currents must arise predominantly from bacteria not attached to the gate.

Indeed, EIS measurements taken in M9 solution with lactate (80 × 10^−3^ m) before the addition of bacteria, after ≈3 h of chronoamperometric attachment with bacteria at OD_600_ = 1.56, and ≈3 h after exchanging the solution to M9 and lactate (80 × 10^−3^ m) showed a signature of increased anodic reactions, typically observed for MFCs with *S. oneidensis*,^[^ ^38^, ^39^ ^]^ only for the solution still containing an excess of free flowing bacteria (Figure 2d,e). All EIS measurements were carried out at *V*_G_ = +0.3 V_Ag/AgCl_ to maintain bacterial EET. The mentioned signature is manifested as a bending of the Nyquist plot toward the real axis of the impedance at low frequencies (Figure 2d), associated with an upward phase shift of the impedance toward 0° below 1 Hz (Figure 2e). This behavior indicates a transition from the typical high‐impedance capacitive charging of the gate electrode at low frequencies toward a more resistive behavior, due to available exchange current at the electrode from lactate metabolism coupled to EET.^[^ ^38^ ^]^ The upward phase shift could not be observed in the abiotic experiments, neither when bacteria were present on the gate only (Figure 2d,e). To enhance the EET, we added riboflavin in its oxidized form (Rb−) to the solution, which is recognized by the bacteria as a soluble electron acceptor and is known to greatly enhance metabolic currents of *S. oneidensis* MR‐1 already at the concentration of 10 × 10^−6^ m.^[^ ^17^ ^]^ However, 10 × 10^−6^ m Rb(−) did not trigger any measurable current response when the bacteria were attached to the gate only (Figure 2c), as well as no signature of EET in EIS measured ≈3 h after the addition of Rb(−) (Figure 2d,e). The most plausible explanation for these results is that the amount of bacteria present at the gate was not adequate to convert enough Rb(−) into the reduced form Rb(+) that then can be re‐oxidized at the gate to produce a measurable current. Importantly, these experiments revealed a serious limitation of classical amperometry and EIS in the monitoring of EET for a limited number (≈2 × 10^4^) of exoelectrogenic bacteria confined to a small region.

Next, we connected all three terminals of the transistors (source, drain, gate, Figure S6, Supporting Information), transforming the devices into operating OMECTs. To make sure that the attachment process of bacteria did not hinder the working of the OECT, we calculated the transconductance (*g*_m_) after measuring transfer curves, see Figure S7a,b (Supporting Information), respectively. The transconductance is a figure of merit of OECTs, and corresponds to the extent of amplification of the effective gate‐source voltage (*V*_GS_) to the channel current (*I*_DS_) (*g*_m_ = ∂*I*_DS_/∂*V*_GS_). There were no noticeable changes in *g*_m_ values attributable to bacteria for the polarized “bacteria gates” before and after the chronoamperometric attachment. Rather, as comparisons with abiotic devices have shown, a slight decrease in *g*_m_ is systematically observed after a long chronoamperometric process (Figure S8a,b, Supporting Information). This decrease in performance, negligible for the OMECT operation, was also evident from the output curves (Figure S9, Supporting Information). The highest transconductance value of the OMECT of 4.3 mS, recorded at *V*_GS_ = +0.3 V, should provide a substantial amplification of *I*_GS_, allowing lactate detection with relatively low number of bacteria accommodated by the microfabricated gate.

For measuring EET, we operated OMECTs at *V*_GS_ = +0.3 V and *V*_DS_ = −0.3 V under constant nitrogen purging in M9 buffer. Addition of lactate into the solution (80 × 10^−3^ m) resulted in a clear current response for both the PEDOT:PSS and PEDOT:PSS‐PVA devices where bacteria were attached on the gate, with no free flowing cells in the electrolyte (**Figure** **3**a). For every electron transferred by bacteria to the positively polarized gate, a positive ion goes from the electrolyte into the channel and charge‐compensates a negative PSS^−^. For each compensated PSS^−^, a hole is extracted from the channel. This effect results in a de‐doping (reduction) of the PEDOT phase within the PEDOT:PSS and PEDOT:PSS‐PVA transistor channels, thus a decrease in the *I*_DS_ current, upon lactate addition (response curves, Figure S10a,b, Supporting Information). Δ*I*_DS_/*I*_DS_, representing the change in signal relative to the level before addition of lactate, for PEDOT:PSS‐based devices increased constantly over the period of monitoring (≈50 min), while for PEDOT:PSS‐PVA‐based devices it saturated after ≈40 min from the addition of lactate, reaching 0.4% and 1.6%, respectively. This temporal response to lactate by *S. oneidensis* MR‐1 is an order of magnitude faster compared to recording by classical chronoamperometry either with an excess of bacteria (Figure 2a) or with biofilms grown on bulky electrodes, the latter commonly reported in the literature.^[^ ^17^, ^40^ ^]^ In contrast to the OMECTs, the transient lactate responses of both abiotic devices were small, with Δ*I*_DS_/*I*_DS_ gradually receding back to the background level (Figure 3a). Based on these control experiments, we can exclude electrochemical reactions of lactate as the origin of the change in *I*_DS_. We also note that attempts to quantify EET in oxygenic environment was hindered by the strong response of OECTs to oxygen via the oxygen reduction reaction (ORR; see more details in Figure S11 in the Supporting Information). In addition, we neglect any possible electrostatic interactions between the negatively charged bacterial cell wall and the active materials of the transistor as the source of recorded signals shown in Figure 3. This is because we expect the relatively high NaCl concentration in our electrolyte (≈10 × 10^−3^ m) to electrostatically screen any such interactions.^[^ ^27^ ^]^

**Figure 3:**
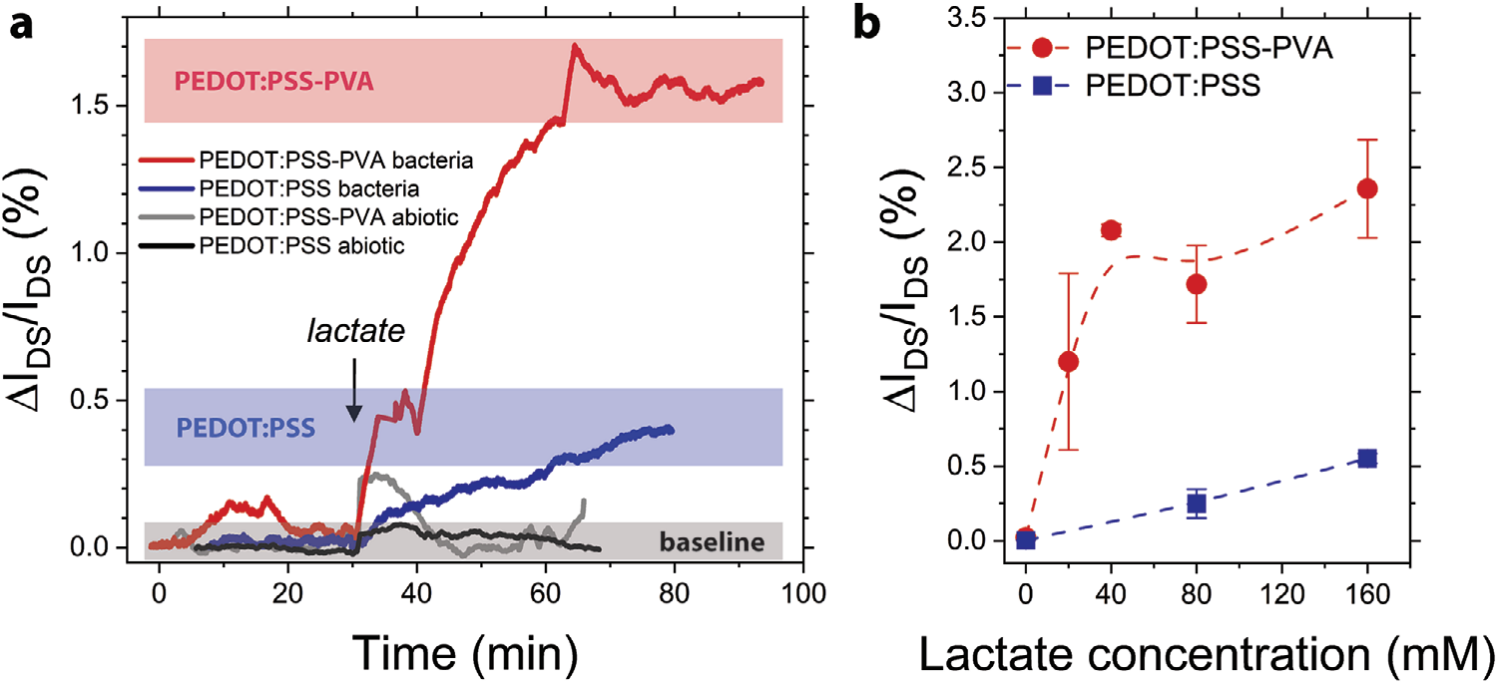
Amplification of EET via OMECTs. a) Normalized current response curves of PEDOT:PSS (blue = biotic; black = abiotic) and PEDOT:PSS‐PVA (red = biotic; gray = abiotic) devices versus time to addition of 80 × 10^−3^ m lactate (black arrow). The red, blue and gray bars are visual guides to signal levels at the end of monitoring time of biotic PEDOT:PSS‐PVA and PEDOT:PSS, and abiotic devices, respectively. b) Normalized current response curves of OMECTs versus different lactate concentrations. Error bars represent the standard error of mean from three (PEDOT:PSS) and four (PEDOT:PSS‐PVA) measurements, each based on a different device. The time interval between each lactate addition was 60–90 min. Data points were determined each time right before addition of new lactate.

Further, a set of experiments with different lactate concentrations indicated good reproducibility, and also consistently stronger response for PEDOT:PSS‐PVA OMECTs as compared to the PEDOT:PSS devices (Figure 3b). The same set of experiments also revealed an increasing trend in the lactate response with lactate concentration in the range of 20–160 × 10^−3^ m for both types of devices, suggesting that the OMECT platform has a potential to capture very subtle differences in EET response from a small number of bacteria. Finally, the advantage of amplifying weak electrochemical signals induced by electrogenic bacteria at the gate of OECTs was further demonstrated by no detectable changes in *I*_GS_ upon lactate addition (Figure S10a,b, Supporting Information). To the best of our knowledge, these results constitute the first amplification of EET from bacteria via microfabricated transistors which also reveal an unexpectedly fast response of *S. oneidensis* MR‐1 to lactate on the order of minutes rather than the commonly observed time span of several hours.

In conclusion, we successfully integrated microfabricated OECTs with the exoelectrogenic bacteria *S. oneidensis* MR‐1 by depositing the bacteria onto the 0.25 mm^2^ transistor gate. A chronoamperometric attachment process performed in a deoxygenated environment revealed that excess cells in the electrolyte create metabolic currents from lactate oxidation. Although fluorescence microscopy images confirmed a good coverage of the gate area by bacteria, we were not able to obtain signatures of EET from these microbes attached to the gate only using classical chronoamperometric and EIS techniques, in the presence of lactate and even on addition of external flavin shuttles. Finally, we succeeded in harnessing and monitoring EET resulting from the metabolism of attached bacteria to microfabricated gate electrodes through the amplification effect of the OECTs via the de‐doping process of the PEDOT transistor channel. In addition, we have shown that inclusion of PVA into PEDOT:PSS yields overall improved results, presumably due to enhanced attachment of bacterial cells to the surface of PEDOT:PSS. The OMECT platform also revealed an order of magnitude faster EET response of *S. oneidensis* MR‐1 to lactate compared to studies using classical electrochemical approaches on bulky charge collectors. Our work is a first step toward investigating and monitoring EET in highly confined spaces via microfabricated organic electronic devices, and our findings may be of importance in studying exoelectrogens in microenvironments, such as the human microbiome.^[^ ^9^ ^]^

## Experimental Section

##### Formulation of Solutions for the Conducting Polymeric Films

PEDOT:PSS (Heraeus, Clevios PH1000) was used as the conducting polymer active layer. 50 µL mL^−1^ ethylene glycol (average *M*_W_ 62.07 g mol^−1^, Sigma Aldrich) and 0.5 µL mL^−1^ dodecylbenzenesulfonic acid (DBSA, average *M*_W_ 326.49 g mol^−1^, Sigma Aldrich) were added to the PEDOT:PSS dispersion to increase the conductivity and improve the film structure. A 10 wt% solution of PVA (average *M*_W_ 130 000, Sigma Aldrich) was prepared by sonicating a PVA:deionized water mixture until a clear solution was obtained. 25 µL mL^−1^ of 10 wt% PVA was later added to the above PEDOT:PSS dispersion followed by sonication for 1 h. In the final step, 0.25 wt% (3‐glycidyloxypropyl)trimethoxysilane (GOPS) (*M*_w_ 236.34 g mol^−1^, Sigma Aldrich) was added to improve the adhesion of the polymeric film to glass substrates. For the PEDOT:PSS solution not containing PVA, 1 wt% GOPS was added instead. The above mixture was then sonicated again for 30 min before being filtered through a 0.45 µm PVDF filter to avoid aggregates.

##### Device Fabrication

The process, similar to that reported previously,^[^ ^25^ ^]^ included the deposition and patterning of Au, Pa‐C, and PEDOT:PSS or PEDOT:PSS‐PVA. Source‐drain contacts were patterned by a lift‐off process with an S1813 photoresist exposed to UV light through a Karl‐Suss mask aligner and developed with an MF‐26 developer. Chromium (2 nm) and Au (50 nm) were subsequently deposited by vacuum evaporation, and metal lift‐toff was carried out in acetone and isopropanol. Metal interconnects and pads were insulated by the deposition of 2 µm Pa‐C with a Diener parylene coater, with a silane A‐174 adhesion promoter used during deposition. A dilute solution of 2% industrial cleaner (Decon‐90) in deionized water was subsequently spin‐coated to act as an antiadhesive layer for a second, sacrificial 2 µm Pa‐C film. Samples were subsequently patterned with a 7.5 µm thick layer of m‐ap 1275 photoresist and developed with the corresponding developer. The patterned areas were opened by reactive ion etching with an oxygen plasma (Oxford Plasma). PEDOT:PSS or PEDOT:PSS‐PVA solutions were spin‐coated at 3000 rpm and baked for 90 s at 100 °C.^[^ ^29^ ^]^ The second layer of Pa‐C was peeled off with a subsequent rinsing in deionized water and baking at 140 °C for 45 min. To form the well/reservoir for the M9 electrolyte‐bacteria‐lactate solution polyacrylate pipes were cut into dimensions of 3 cm (height) and 2 cm (diameter). These wells were glued onto the Pa‐C‐coated glass substrates using PDMS followed by thermal crosslinking at 80 °C for 15 min. Sylgard 184 Silicone Elastomer kit was used to prepare the PDMS solution by mixing the oligomer (silicone elastomer base) and a radical initiator (curing agent) in the ratio of 5:1, respectively. A centrifuge system was used to de‐gas the resultant mixture.

##### Strains and Growth Conditions

All strains used in these experiments were derived from wild type *S. oneidensis* MR‐1. The majority of experiments were performed using a GFP‐expressing strain, which carries a plasmid encoding *gfp* and a kanamycin‐resistance gene, and enables visualization by fluorescence microscopy.^[^ ^33^ ^]^ The Δ*mtrB* mutant lacks the transmembrane porin MtrB and is therefore unable to carry out EET.^[^ ^41^ ^]^ Cultures were inoculated from frozen glycerol stocks into two 50 mL sterile falcon tubes, each containing 10 mL Luria‐Bertani (LB, Sigma‐Aldrich) broth and grown overnight at 30 °C with 250 rpm shaking to OD_600_ = 2.1 and 3.9 for the GFP‐and Δ*mtrB* mutants, respectively. After overnight growth the cells were harvested by centrifugation at 4000 RCF at 4 °C for 10 min and washed and resuspended twice with M9 Minimal Salts medium (M9, Sigma‐Aldrich). The first washing was followed by one more centrifugation step with the same parameters as above. Finally, the combined cell pellet was resuspended in 2 mL M9 medium resulting in tenfold concentrated stock suspensions. Bacteria were handled under sterile conditions. Cultures of *S. oneidensis* MR‐1 expressing GFP were grown with 50 µg mL^−1^ kanamycin (Sigma Aldrich). Optical density values were determined using a Lambda 900 (PerkinElmer) absorption spectrometer by 20‐fold dilution of stock suspensions in M9 medium using quartz cuvettes with 1 cm optical pathlength. It was noted that the growth rates for the two mutants differed, therefore different volumes of the concentrated stock suspensions were added for the experiments shown in Figure 2 to reach the same OD_600_ = 1.56.

##### Electrochemical Techniques

For all experiments an Interface 1010B potentiostat/galvanostat was used controlled by the Gamry Framework software (both from Gamry Instruments). +0.3 V was applied on the gate versus a Ag/AgCl wire, the latter acting as a combined reference and counter electrode. In all cases nitrogen gas was directly purged into the M9 solution containing 80 × 10^−3^ m lactate (Sigma Aldrich). To slow down the evaporation of the electrolyte solution, wet tissues were placed in the close vicinity of devices. EIS was performed with a stimulus of 10 mV peak‐to‐peak on a +0.3 V_Ag/AgCl_ DC voltage in the frequency range from 20 kHz to 10 mHz.

##### Microscopy on the Gate

After chronoamperometric bacteria attachment, devices were gently rinsed with M9 then deionized water, followed by drying using nitrogen gas. Plastic wells were carefully detached from the substrates mechanically prior to imaging. Imaging was done on an Ni‐E upright motorized microscope (Nikon), equipped with a Zyla sCMOS camera (Andor Technology). The illumination and excitation source was a Lambda DG‐4 ultra‐high‐speed wavelength switching illumination system (Sutter Instrument), introduced from the top side. The area of observation was precisely selected by controlling a microscope stage in *X*–*Y* directions using a ProScan III universal microscope automation controller (Prior). Different magnifications were achieved by manually switching between a S Plan Fluor ELWD 20X/0.45 OFN22 DIC N1 and a CFI60 TU Plan EPI ELWD 50X/0.60 infinity corrected objectives (both from Nikon). The microscope was equipped by bright field and FITC filter sets, the latter for fluorescence imaging. In Figure 2b, imaging was done through 20X (top, exposure times 2 and 5 s, respectively) and 50X (bottom, exposure times 5 s for both devices) objectives. The number of cells on the PEDOT:PSS gate was estimated from the grayscale 16‐bit image not containing the scale bar, corresponding to Figure S1 (Supporting Information), using ImageJ (Fiji)^[42,43]^ by the following process: the area outside of the gate was excluded; then, intensity threshold values were set so that most of bacteria were visible, followed by converting the grayscale colors into black and white binary colors; after that the resulting image was segmented using the “Watershed” algorithm; finally, the number of cells were counted using “Analyze Particles” method, while particles below the size of 0.65 × 0.13 µm^2^ (corresponding to the area of 5 pixels on the image obtained using a 50X objective) were not counted. The number of bacteria were multiplied by 3, as the analyzed region shown in Figure S1 (Supporting Information) constitutes one‐third of the total gate area.

##### OECT Characterization and Lactate Detection via OMECTs

OECT devices were characterized using a Keithley 2600 series source measure unit (SMU) and controlled using a customized LabVIEW program. All response curve measurements were carried out in M9 electrolyte with concentrations of lactate in the range of 20–160 × 10^−3^ m. Before the attachment process of bacteria on OECTs, each device was stabilized with pulsed voltage at the gate from *V*_GS_ = 0 V to *V*_GS_ = +0.5 V with *V*_DS_ fixed at −0.5 V. After attachment of bacteria, OMECTs were operated at constant *V*_DS_ = −0.3 V and *V*_GS_ = +0.3 V under constant N_2_ purging (0.5 bar) into M9, for the lactate response measurements. After stabilization of the channel current (*I*_DS_), lactate was added to the M9 solution. The time interval between each lactate addition was 60–90 min. This was to allow sufficient time for the *I*_DS_ current to stabilize, which in turn enabled better normalized EET readouts. Each set of response curve data, where one set consisted of measurements in the lactate range of 20–160 × 10^−3^ m, was carried out using one OMECT device, while the data points shown in Figure 3b were determined from *I*_DS_ 60–90 min after the addition of lactate.

## Conflict of Interest

The authors declare no conflict of interest.

## Supporting information

Supporting Information
