# Supplementary material for: Organic Microbial Electrochemical Transistor Monitoring Extracellular Electron Transfer
Source: Adv Sci (Weinh). 2020 Jun 9;7(15):2000641. doi: 10.1002/advs.202000641 (PMC7404149; doi:10.1002/advs.202000641)
Supplement: Supplementary file 1 — Supporting Information [file ADVS-7-2000641-s001.pdf]

© 2020 Wiley-VCH GmbH

ADVANCED SCIENCE
Open Access

Supporting Information

for *Adv. Sci.*, DOI: 10.1002/advs.202000641

Organic microbial electrochemical transistor monitoring extracellular electron transfer

Gábor Méhes[1a], Arghyamalya Roy[1a], Xenofon Strakosas[1a], Magnus Berggren[1,2], Eleni Stavrinidou[1,2]* and Daniel T. Simon[1,2]*

SUPPORTING INFORMATION

Organic microbial electrochemical transistor monitoring extracellular electron transfer

Gábor Méhes[1a]*, Arghyamalya Roy[1a], Xenofon Strakosas[1a], Magnus Berggren[1,2], Eleni Stavrinidou[1,2]* and Daniel T. Simon[1,2]

[1] Laboratory of Organic Electronics, Department of Science and Technology, Linköping University, Norrköping, Sweden

[2] Wallenberg Wood Science Center, Department of Science and Technology, Linköping University, Norrköping, Sweden

[a] Authors contributed equally

* Corresponding authors: eleni.stavrinidou@liu.se, gabor.mehes@liu.se and mehes.g@gmail.com

Contents

1. Description of OMECT operation under alternating nitrogen and oxygen gas purging

2. Figures S1-S11

1. Description of OMECT operation under alternating nitrogen and oxygen gas purging

As shown in Supplementary Figure S11 below, we monitored EET in the transistor configuration while alternately purging nitrogen, then oxygen, then again nitrogen gas into the electrolyte where GFP bacteria were attached on the gate but not present in the solution. Qualitatively we can conclude from this graph that we could see a clear response to lactate when nitrogen gas was being purged into the solution (change in I_{DS}), both before and after a period of saturating the solution with oxygen gas. This observation suggests that the bacteria are ‘tolerant’ to oxygen in terms of repeatedly carrying out EET in anoxygenic conditions, when the period of oxygenic condition was 100 minutes or longer.

In addition, as it is obvious from Figure S11, oxygen purging has resulted in a very strong response from the OECT that we assume is from oxygen reduction reaction (ORR). ORR occurs at the channel because it is negatively biased (V_{DS} = -0.3V and V_{GS} = +0.3V), and PEDOT exhibits high efficiency for ORR (cf. doi:10.1039/C6TA10521A and doi: 10.1021/acs.jpcc.7b03210). ORR however, induces an opposite but much more intense change in I_{DS} compared to EET. This effect, caused by oxygen, is hindering the observation of EET during oxygenic condition.

2. Figures S1-S11

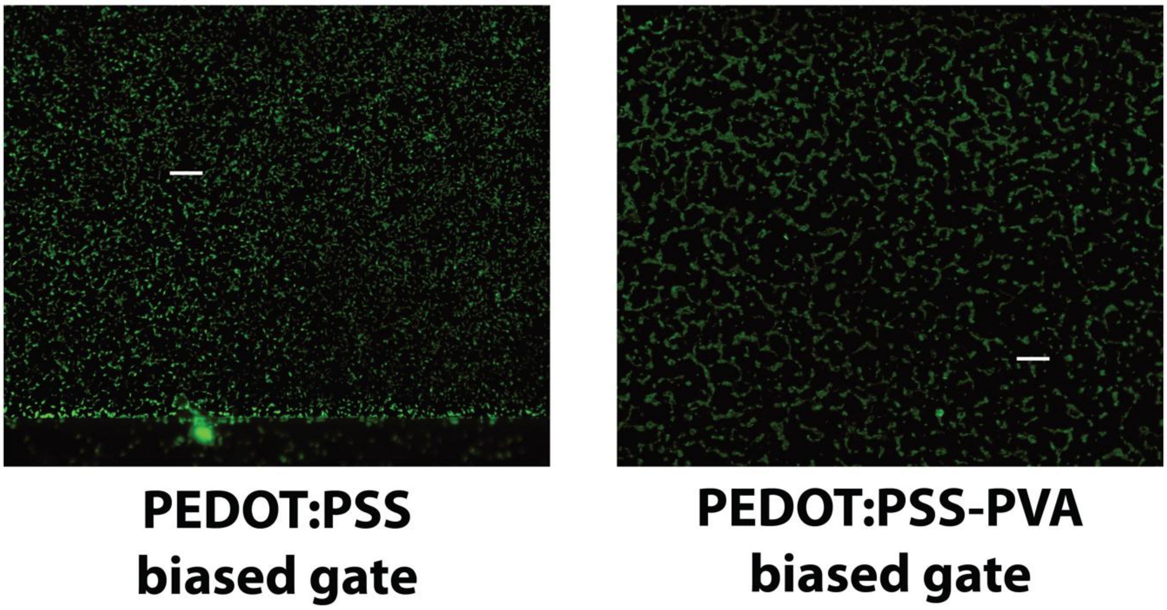

Figure S1. Fluorescence microscopy images of PEDOT:PSS and PEDOT:PSS-PVA polarized gates corresponding to Figure 2b bottom. Scale bars are 20 µm.

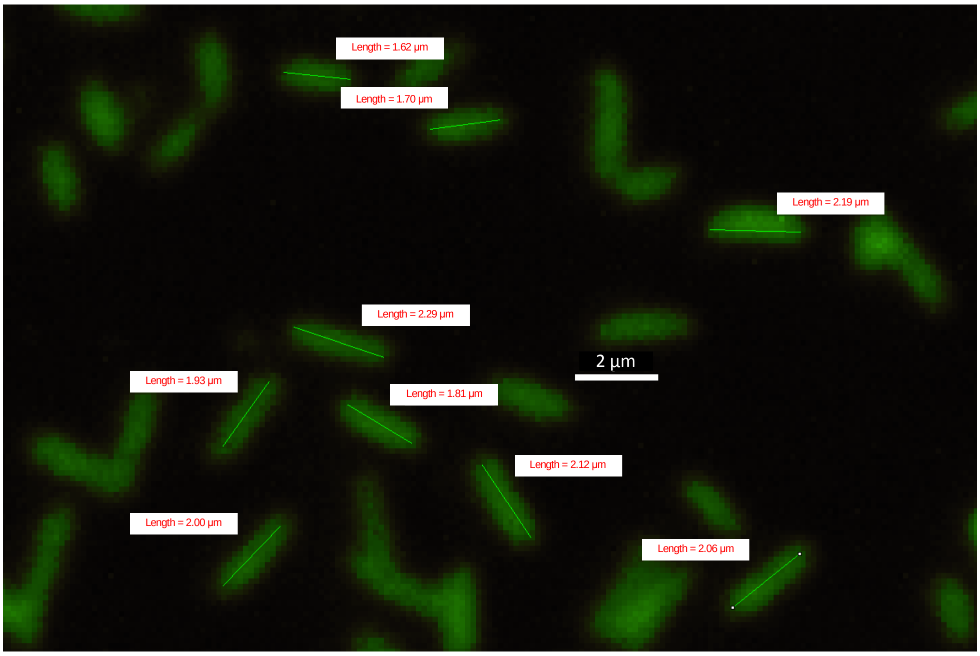

Figure S2. Magnified (digital zoom) microscopy image of a PEDOT:PSS gate after attachment of GFP bacteria observed through a FITC filter. The length measurement of individual bacteria was carried out by the software NIS Elements (Nikon).

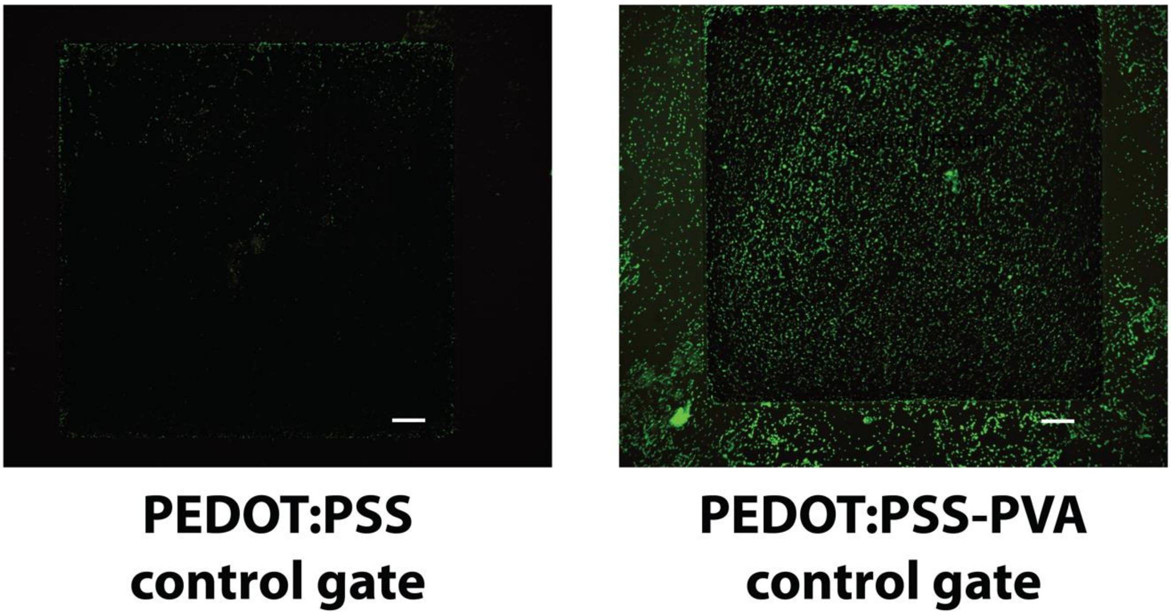

Figure S3. Fluorescence microscopy images of PEDOT:PSS and PEDOT:PSS-PVA non-polarized “control” gates after attachment of GFP bacteria, observed through a FITC filter. Exposure time was 2 s for PEDOT:PSS and 5 s for PEDOT:PSS-PVA. Scale bars are 50 μm.

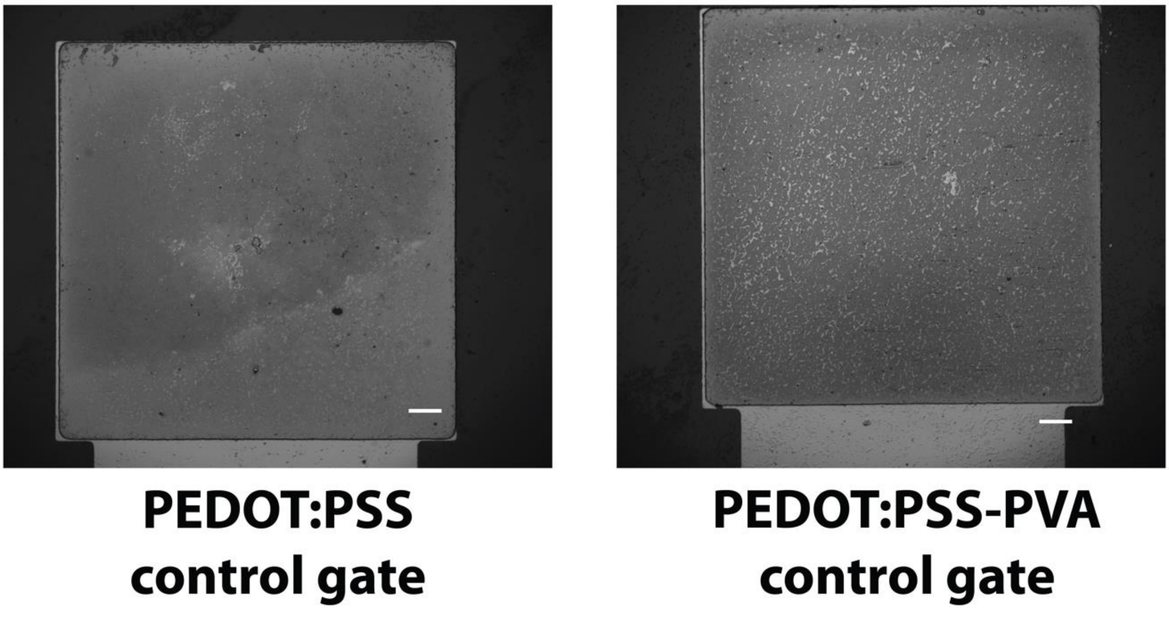

Figure S4. Bright-field microscopy images of PEDOT:PSS and PEDOT:PSS-PVA non-polarized “control” gates after attachment of GFP bacteria, corresponding to Supplementary Figure S2. Exposure time was 20 ms for both devices. Scale bars are 50 μm.

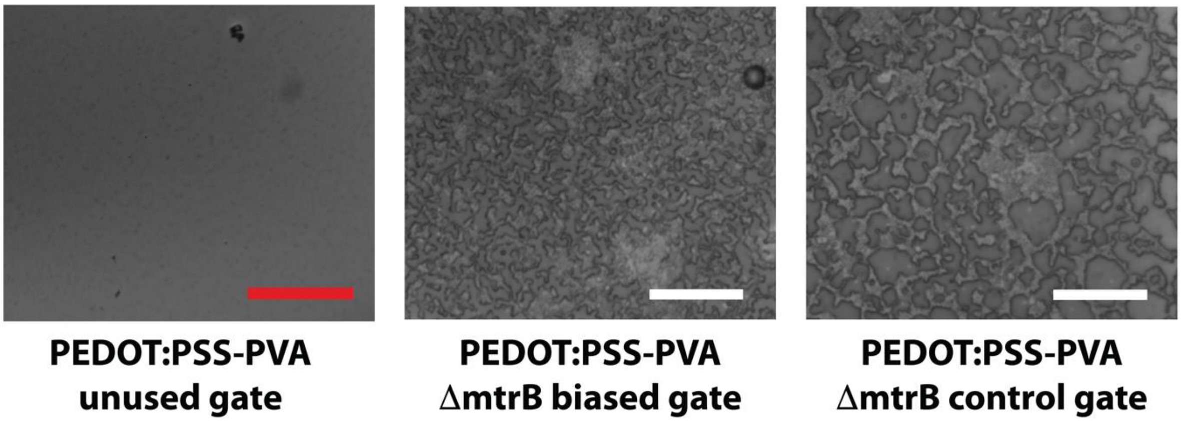

Figure S5. Bright-field microscopy images of PEDOT:PSS-PVA "unused" gate before attachment, polarized (biased) and non-polarized "control" gates after attachment of Δ*mtrB* bacteria. Red and white scale bars are 50 and 20 μm, respectively.

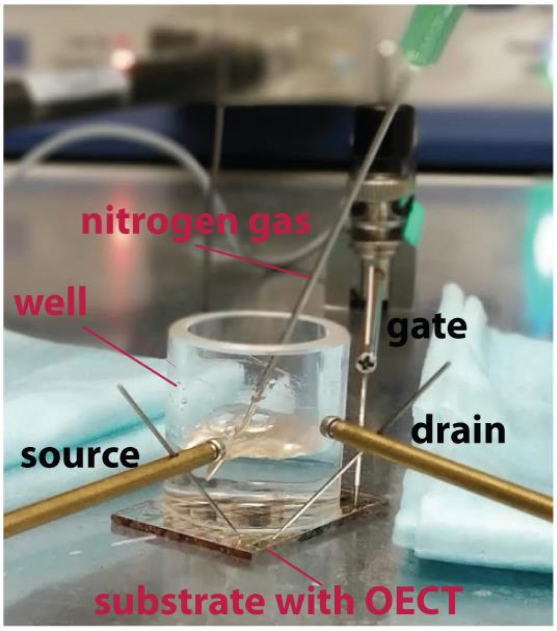

Figure S6. Photographic snapshot of an OMECT with electrical connections.

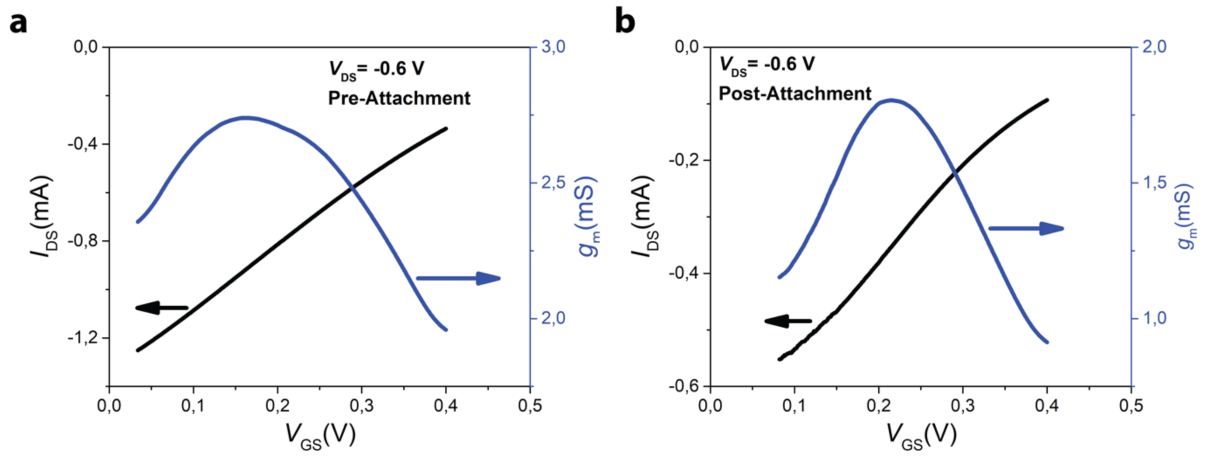

Figure S7. Representative transconductance- and corresponding transfer curves (blue and black lines, respectively) of an OMECT based on a PEDOT:PSS-PVA device a) before and b) after the chronoamperometric attachment, measured at V_{DS}= -0.6 V.

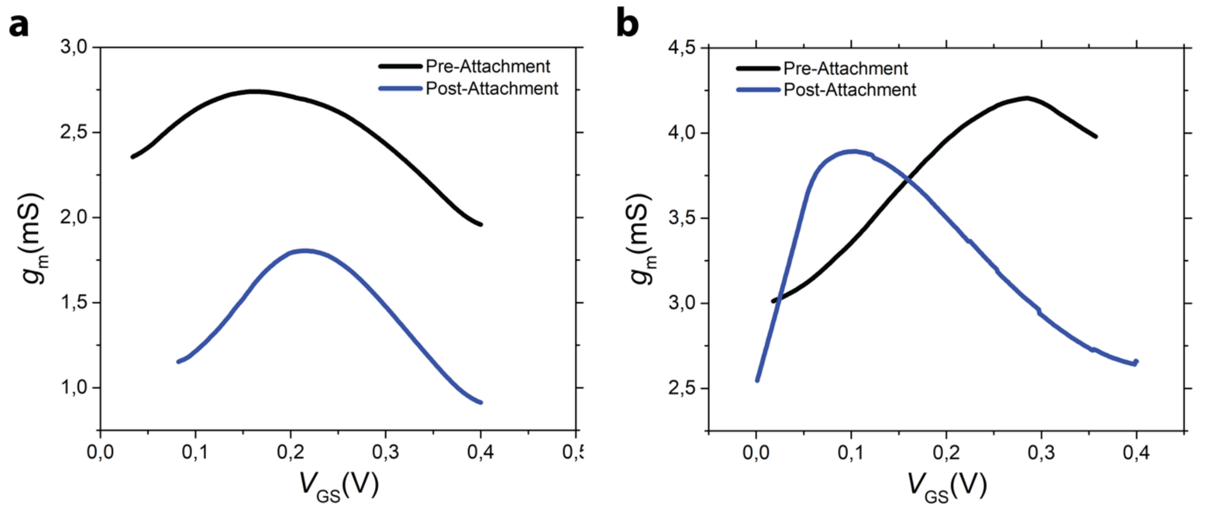

Figure S8. Representative transconductance curves of a) an OMECT and b) an abiotic OECT for PEDOT:PSS-PVA devices before and after the chronoamperometric attachment (black and blue lines, respectively).

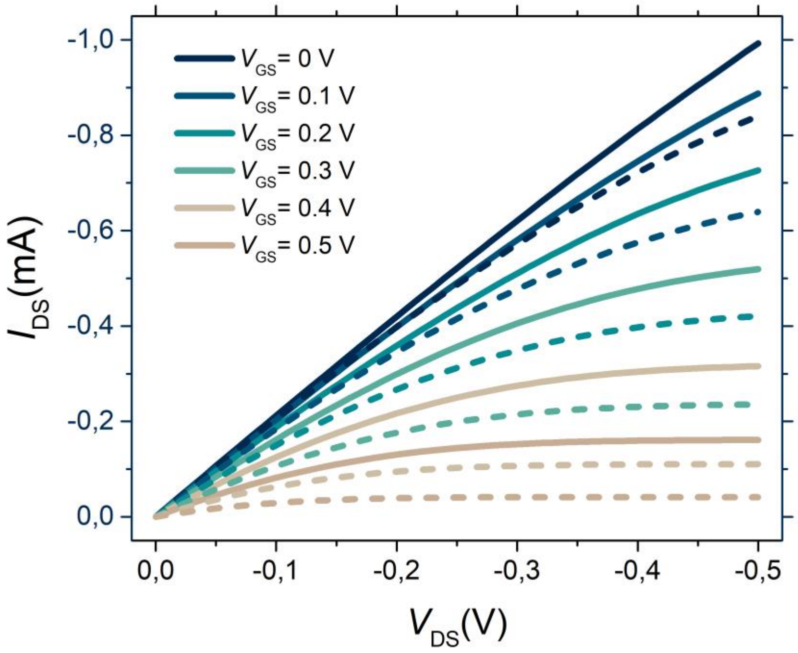

Figure S9. Representative output curves of OMECTs for a PEDOT:PSS-PVA device before (solid lines) and after (dashed lines) the chronoamperometric attachment for various V_{GS} conditions.

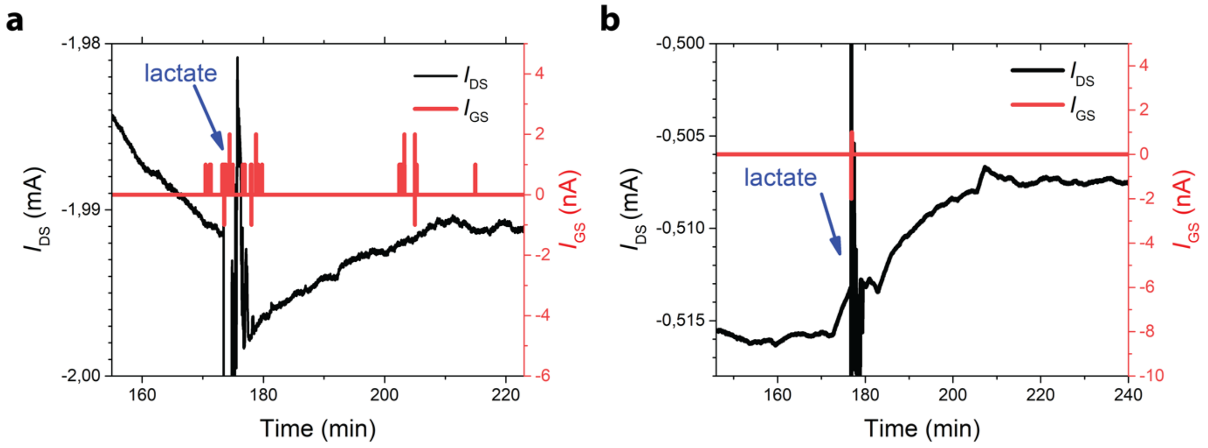

Figure S10. Response curves of I_{DS} (black lines) and I_{GS} (red lines) upon lactate addition (80 mM) for a) PEDOT:PSS and b) PEDOT:PSS-PVA OMECTs presented in Figure 3a.

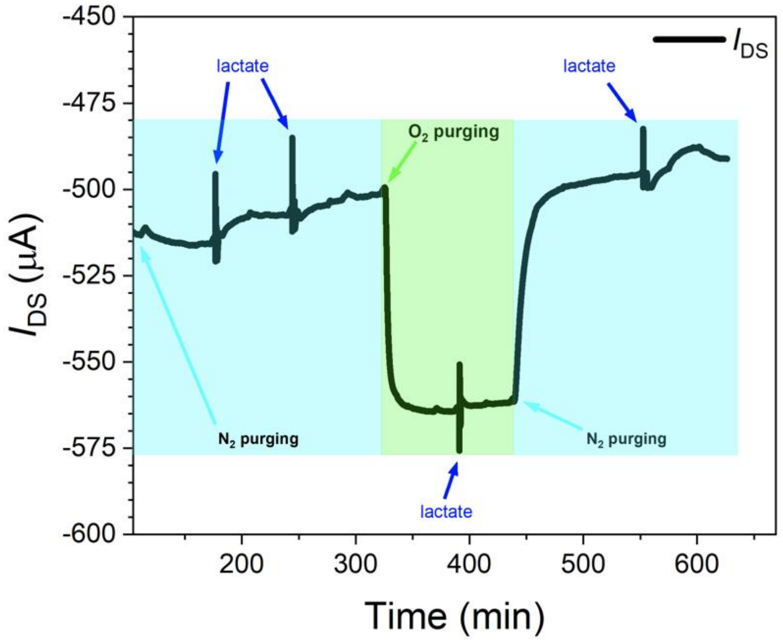

Figure S11. Response curve of I_{DS} upon lactate additions (80 mM each time) and alternating nitrogen/oxygen gas purging for a PEDOT:PSS-PVA OMECT at constant V_{DS}= -0.3 V and V_{GS}= 0.3 V, GFP mutant present on the gate.
